# Exploring the Concept of Degrees of Maternal Morbidity as a Tool for Surveillance of Maternal Health in Latin American and Caribbean Settings

**DOI:** 10.1155/2017/8271042

**Published:** 2017-10-22

**Authors:** Suzanne J. Serruya, Bremen de Mucio, Gerardo Martinez, Luis Mainero, Andres de Francisco, Lale Say, Maria H. Sousa, Renato T. Souza, Maria L. Costa, Jussara Mayrink, Jose G. Cecatti

**Affiliations:** ^1^Latin American Centre of Perinatology (CLAP-WR/FGL-PAHO), Montevideo, Uruguay; ^2^Pan American Health Organization (PAHO/FGL), Washington, DC, USA; ^3^World Health Organization (WHO), RHR/HRP, Geneva, Switzerland; ^4^Department of Obstetrics and Gynecology, University of Campinas, Campinas, SP, Brazil

## Abstract

**Objectives:**

To assess a birth registry to explore maternal mortality and morbidity and their association with other factors.

**Study Design:**

Exploratory multicentre cross-sectional analysis with over 700 thousand childbirths from twelve Latin American and Caribbean countries between 2009 and 2012. The WHO criteria for maternal morbidity were employed to split women, following a gradient of severity of conditions, into (1) maternal death (MD); (2) maternal near miss (MNM); (3) potentially life-threatening conditions (PLTC); (4) less severe maternal morbidity (LSMM); (5) any maternal morbidity; and (6) women with no maternal morbidity. Their prevalence and estimated risks of adverse maternal outcomes were assessed.

**Results:**

712,081 childbirths had a prevalence of MD and MNM of 0.14% and 3.1%, respectively, while 38% of women had experienced morbidity. Previous maternal morbidity was associated with higher risk of adverse maternal outcomes and also the extremes of reproductive ages, nonwhite ethnicity, no stable partner, no prenatal care, smoking, drug and alcohol use, elective C-section, or induction of labour. Poorer perinatal outcomes were proportional to the severity of maternal outcomes.

**Conclusions:**

The findings corroborate WHO concept regarding continuum of maternal morbidity, reinforcing its importance in preventing adverse maternal outcomes and improving maternal healthcare in different settings.

## 1. Introduction

In 2009, after decades of using maternal mortality as the most important health indicator for women experiencing the reproductive process, the definition of maternal near miss (MNM) with its correspondent criteria was published. Women suffering a severe complication during pregnancy, childbirth, or within 42 days of the postpartum period, who almost died, but survived due to luck or effective interventions are now considered* maternal near miss* [1].

The need for better exploring the concept of maternal morbidity instead of maternal mortality arose because, fortunately, maternal deaths became rare in several settings when using absolute numbers and, therefore, it became more difficult to understand and identify factors or conditions that could possibly be associated with its occurrence. There are also a number of reasons already publicized on the advantages of looking into cases of morbidity and not only to maternal deaths [2]. The most important ones refer to the fact that if the morbidity is timely identified and properly managed, actions or interventions can be performed and the death and organ failure could potentially be avoided. In addition, the woman surviving and being alive could help a lot with information on the entire process that she experienced, including the delays and difficulties she had for having access to appropriate care.

However, until recently, the concept of maternal morbidity and the levels of severity it could present were definitely not standardized, and therefore they were used indistinctly in the scientific literature with different meanings. This, of course, represented a restriction for using the concept as a real health indicator that could be used for policy changes or even as a starting point for interventions and for comparisons between different settings or in the same location across different periods. With the WHO definition and criteria [1], theoretically, this is no longer a restriction. Notwithstanding the problem is not yet completely solved since the criteria for potentially life-threatening conditions and maternal near miss are still not officially adopted everywhere and the correspondent routine data is not systematically collected for surveillance purposes all over the world.

Taking these limitations into account and the availability of several possible sources of information on maternal morbidity, a considered approach was to build a pragmatic definition of maternal near miss that could be even retrospectively applied to obtain relevant information on the condition, in order to direct next steps for policy changes.

The opportunity came with the information already collected from the WHO Global Survey on Maternal and Perinatal Health. Several different combinations of conditions that were hypothesized as possible predictors of maternal death due to its severity were tested in their predictive capacity of identifying cases of women who died from a maternal cause. The group of most common criteria with the highest accuracy for this prediction included hysterectomy due to haemorrhage or infection, admission to intensive care unit (ICU), blood transfusion, and eclampsia. Altogether, they are now the pragmatic criteria established by the WHO [3].

Although already officially recommended by WHO for gathering information on maternal morbidity and also for appraising the quality of maternal healthcare [1, 4], these criteria are not yet included in routine data collection on care during childbirth in the majority of countries. The knowledge already available on these conditions comes basically from some important national and international studies that are of course episodic and do not constitute a systematic surveillance system applied to routine care [5–9]. Currently WHO is doing an effort to conceptualize, classify, and define criteria for other less severe maternal morbidities and trying to develop and test specific ways and tools for measuring the burden of these conditions on the life of women [10–12]. Therefore, there is still a need for a better understanding of complications profile in several different settings and scenarios on the occurrence of maternal morbidity, the role that severe cases play in maternal health services and the real capacity of improving the quality of care when a severe condition is timely identified during the process of morbidity.

Keeping these points into account, the objective of the current study is to perform an exploratory analysis of the database on maternal and neonatal information for childbirths occurring in several maternity hospitals located in the Latin America and Caribbean region and coordinated by CLAP, the Latin American Centre for Perinatology, Women and Reproductive Health from the Department of Family, Gender and Life Course of PAHO. The product of this analysis may be useful for identifying a group of severity markers taken as a proxy or pragmatic criteria for levels of severity in maternal morbidity and as well for building a general profile of maternal morbidity for Latin America and the Caribbean region.

## 2. Methods

Information on childbirth in the region of Latin America and the Caribbean, for both maternal and neonatal health conditions, has been routinely stored in the SIP (Perinatal Information System) database, when shared by countries or health institutions, during more than 25 years [13]. Since the system started working in 1983, countries and health facilities from the region can volunteer for using it, filling the standard forms developed for collecting information on pregnancy, childbirth, and neonatal conditions, after staff had been trained. The system is free of charge and currently works on a web-based platform, enabling generation of institutional, country, or regional reports, but only under their specific request. Therefore, some problems as a cohort effect due to changing practices and incompleteness of some variables are possible in the database and represent some of its limitation.

Therefore, although the full SIP database includes more than 4 million records, for the specific objectives currently addressed we assessed and analysed a more recent SIP database containing standardized information on over 700 thousand childbirths which occurred between 2009 and 2012. In this database, there is information from childbirths which occurred in some health facilities from 12 countries from the South Cone, Andean, Central America, and Caribbean subregions from America (Argentina, Bolivia, Colombia, El Salvador, Ecuador, Guatemala, Guiana, Honduras, Haiti, Nicaragua, Paraguay, and Uruguay). The sample for each country does not necessarily represent all births in the country for the period nor is proportional to the population size and therefore is not supposed to be representative of the respective country. This is the reason why the data does not allow for analysis of specific country's reports. With data routinely collected from all births in the period occurring in the participating health facilities, the methodological approach of the current exploratory analysis is that of a cross-sectional study. No sample size was previously estimated because of the huge number of women with data available, although this was not a population-based study. Missing information for variables used was assumed to be randomly distributed among centres, countries, and time and associated neither with predictors nor with outcomes. Although the system used for data collection was built to immediately check for internal consistency and also those in charge of feeding the online platform with data from health facilities received training following standardized operating procedures and instructions, for additional quality control, several cross-checking instances between variables of interest were performed for assessing the consistency of the database. This was performed to improve the quality of data collected. However, of course, the data refers only to cases from centres that voluntarily applied for using the system. No information at all is available for childbirths occurring at home, at the health centre, or at any other health facility not using the system.

This period was chosen because data were gathered with a data collection form standardized for all participating facilities and countries and because this is the period immediately before the introduction of some specific information for identifying maternal near miss cases with the WHO definition and criteria [1]. The main idea was to test the possibility of retrospectively assessing big birth registries to identify different levels of severity of maternal morbidity, thus contributing to policy changes for improving the quality of obstetrical care provided to women, and also the relationship with adverse perinatal outcomes which could have a positive impact on the care of neonates as well. However, we do know that the clinical forms were not built with the aim of identifying a gradient of severity of conditions, but instead for capturing all diagnosis and conditions understood as complications occurring during the childbirth process.

In order to achieve the objectives of identifying different levels of severity for maternal morbidity, in the current analysis as below outlined, we built the groups using some possible identifiers from the database:

1. Group of maternal death (MD): with the purpose of identifying the cases of maternal deaths, this group included the women with information that death had occurred at the health facility or who died during transport or in the place they were transferred to. This was used descriptively to estimate maternal mortality ratio in the sample, however with no classification and no attribution of cause of maternal death because this information was not reliably available in the database.
2. Group of maternal near miss (MNM): to identify the cases with more severe conditions of maternal morbidity that could serve as a proxy for maternal near miss (supposedly with an organ dysfunction or failure), this group (MNM) was identified using some pragmatic criteria already described and even developing a new set of criteria within the database. This was the group with greater difficulty to be identified. According to the WHO pragmatic criteria as a proxy for MNM [3], there were only two specifically recorded in the database: blood transfusion (any amount) and haemorrhage at any trimester or postpartum haemorrhage. We then included also eclampsia or magnesium sulfate used for eclampsia; cardiac disease; renal disease; or prolonged hospital stay (>7 days). This was also used descriptively to build the respective profile of cases of maternal near miss in the sample.
3. Group of potentially life-threatening conditions (PLTC): to identify the cases that could be classified as PLTC in a proxy to these exact conditions as defined by WHO [1]. This group was formed with cases presenting any condition of PLTC defined by WHO as in part A of the attached Box 1 and whose corresponding information is available in specific variables in the SIP database. For this group, the following conditions were taken into account: abruption placentae; ectopic pregnancy; ruptured uterus; postpartum haemorrhage; puerperal sepsis; puerperal infection; severe preeclampsia with no use of magnesium sulfate; severe hypertension; any blood transfusion; placenta previa with haemorrhage; or another severe condition. Again, this information was used descriptively to build the respective profile of PLTC in the sample.
4. Group of less severe maternal morbidity (LSMM): to identify the cases of less severe maternal morbidity, including all other remaining conditions identified as maternal morbidities with specific information collected in the SIP, this group constituted cases presenting any morbidity recorded in the SIP database, other than those above described. For this group, the following conditions were taken into account: anaemia, HIV+, diabetes mellitus, hospital admission during pregnancy, ovular infection, urinary tract infection, another maternal pathological condition, retained placenta, or need to be referred. This was mainly based on the recently issued maternal morbidity matrix, in its dimension one, including symptoms, signs, investigations, and managements related to both direct and indirect causes of maternal morbidity [11]. The profile of this group was described according to some general characteristics of the women and corresponding health indicators were also reported.
5. Group of any maternal morbidity (AMM): to identify all cases of maternal morbidity (joining groups 1 to 4), from the most severe (death) to the less severe ones, this is also aligned with the recent WHO proposal for identifying, classifying, and building a full profile of maternal morbidity occurring worldwide [10, 11]. This group was built by joining all the women included in the previous 4 groups, with any kind of identified maternal morbidity.
6. Group of women with no maternal morbidity: this group represents the remaining women who did not experience any maternal morbidity during childbirth and postpartum period as previously described and survived the event. The remaining women who experienced no identifiable maternal morbidity during the childbirth process were used to build a comparison group for the above-mentioned maternal morbidity and mortality groups, to generate information on factors possibly associated with worse outcomes.

### 2.1. Ethical Issues

The main purpose of the current analysis was to explore the capacity of a big database of a birth registry from Latin America and the Caribbean region to retrospectively identify different levels of maternal morbidity according to what is recently recommended by WHO. It was a methodological exploration approach. The database was not originally built with the objective of an in-depth analysis of maternal mortality and morbidity. Therefore, the analyses allow for neither country variations nor a general and representative scenario of the whole region regarding maternal morbidity. No woman, health facility, or countries were identified and this information is not available in the database. The dataset is regularly fed with the previous agreement of countries that analyses of data are performed at regular intervals. The protocol for the current analyses was previously ethically evaluated and approved by CLAP/WR. All the recommendations of the Declaration of Helsinki for studies involving human beings were strictly followed.

### 2.2. Statistical Analysis

Initially, descriptive analyses were performed, with the prevalence of these adverse maternal outcomes determined together with their correspondent health indicators. Some of these health indicators had already been described and used [1, 5, 7]. Frequencies of each maternal outcome (MD, MNM, PLTC, LSMM, and no morbidity) were stratified according to some sociodemographic factors (maternal age, ethnic group, literacy, and marital status); clinical history (diabetes, hypertension, preeclampsia, eclampsia, other severe medical conditions, cardiac disease, renal disease, and any previous condition); obstetric factors (parity, number of prenatal care visits, and number of caesarean sections); habits (smoking, drugs, alcohol, and/or violence); delivery (year of delivery, onset of labour, and mode of delivery); and perinatal results (gestational age at birth, birth weight, Apgar score at the 5th min, vital status at birth, child condition at discharge, and neonatal near miss (NNM)). The latter, neonatal near miss, corresponds to a composite variable including birth weight below 1750 g, or 5th minute Apgar score <7, or gestational age below 33 weeks [13]. They were then compared to women with no morbidity.

For categorical variables, chi-square tests were used, with *p* values corrected for the cluster design effect (each country was considered as the primary sampling unit (PSU) and then as a cluster). Finally, for the objective of identifying factors independently associated with each degree of the worse maternal outcome, considering the “no morbidity,” the comparison, or reference group, a bivariate and a multiple logistic regression analysis was performed, using all the predictors in the model and considering the PSU in both analyses. These procedures generated estimated crude and adjusted prevalence ratios (PR + 95% CI) for risky categories of these factors in developing maternal morbidity and worse maternal outcomes. For statistical analysis, SPSS v.20.0 and Stata v.7.0 packages were used and the level of statistical significance was of 0.05.

## 3. Results

**Figure 1:**
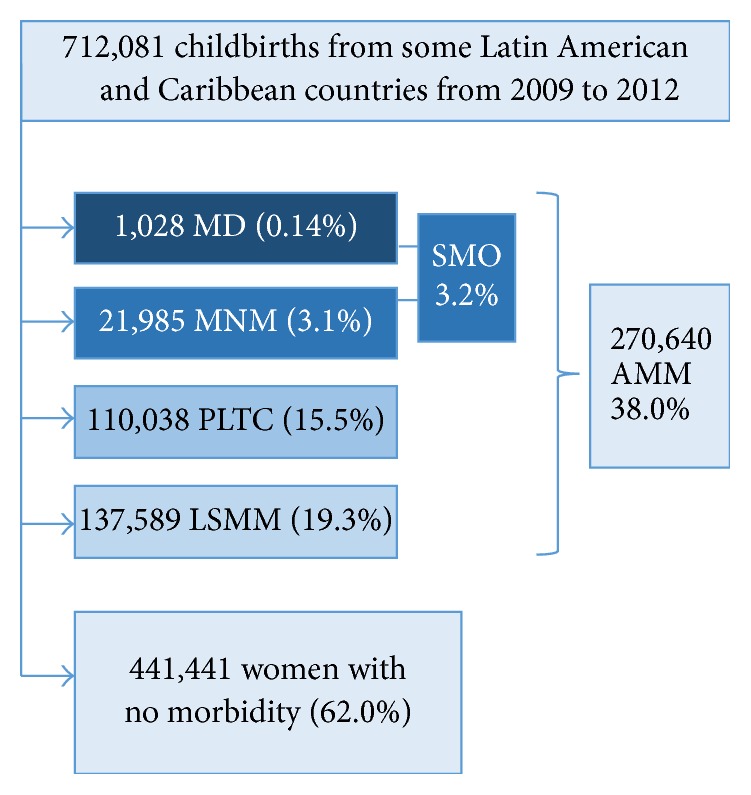
Flow chart of women in the study (AMM: any maternal morbidity; LSMM: less severe maternal morbidity; MD: maternal death; MNM: maternal near miss; PLTC: potentially life-threatening condition; SMO: severe maternal outcomes).

The database from SIP used for the current analysis has information on over 712 thousand women from 12 countries of Latin America and Caribbean region, who were admitted to any health facilities using SIP routinely, for delivery or management of any complication associated with pregnancy. How these women were classified is presented in Figure 1.  Table 1 shows that 1028 maternal deaths were identified in the database, representing 0.14% of women included in the analysis, while maternal near miss occurred in 3.1% of them. The maternal mortality ratio for the sample was as high as 147.3 per 100,000 live births. Conditions classified as PLTC were identified in 15.5% of women and LSMM in 19.3% of them. Therefore, in the sample currently assessed, 38% of women experienced degree of maternal morbidity.

In Table 2, the prevalence of some previous pathological conditions is showed according to the occurrence of adverse maternal outcomes. For all conditions evaluated, including diabetes, hypertension, preeclampsia, eclampsia, cardiac disease, renal disease, other severe condition, or any of the previous conditions, the prevalence significantly increased with the degree of severity, except for cases of maternal death that showed similar proportions as for maternal near miss.

The estimated risks for occurrence of maternal death (MD) were higher for women from ethnic group other than white (indigenous or black people, 19-fold), with any previous pathological condition (2-fold), if labour was induced (1.6-fold) or birth was through an elective C-section (2-fold), as shown in Table 3. On the other hand, these risks were significantly lower for women aged 25–29 years (a 20% lower risk) and for women from mixed ethnic groups (a 65% lower risk). It also shows that the estimated risks of maternal near miss (MNM) were significantly higher for women aged in the extremes of reproductive period, including 10–19 years (13% higher risk) and 35–55 years (39% higher risk), with no a steady partner (23% higher risk), if they are nullipara (32% higher risk), with any previous morbid condition (2.5-fold), with a lower number of prenatal visits (21 to 52% higher risk), for women experiencing smoking, drugs, alcohol, or violence (18% higher risk), if birth occurred with an elective C-section (1.6-fold) or simply by C-section (2.4-fold). A previous C-section was the only condition identified as associated with a reduced risk of MNM (42% lower risk).

Table 4 reports the estimated risks for PLTC, which were higher for women aged above 25 years (an average 5% higher risk), nullipara (14% higher risk), and with any previous pathological condition (43% higher risk). On the contrary, these estimated risks were lower for women with no literacy or schooling until primary level (17% lower risk) and with a previous C-section (34% lower risk). Finally, the estimated risks for LSMM were higher for women aged 10–19 years (10% higher risk), with any previous morbid condition (1.5-fold), and for women experiencing smoking, drugs, alcohol, or violence (28% higher risk), with induced labour or elective C-section (1.3-fold), and delivered by C-section (16% higher risk). On the other hand, these risks were significantly lower for women aged 25–34 years (7-8% lower risk), from the mixed ethnic group, and with no prenatal care (24% lower risk).

Neonatal outcomes stratified by groups of maternal morbidity are reported in Table 5. It shows that, generally speaking, the worse the maternal outcome, the worse the neonatal outcome. In this way, the prevalence of preterm birth, low birth weight, neonatal death at maternal discharge, and neonatal near miss increased significantly with the worsening of the maternal outcome. While neonatal near miss occurred in 4.9% of cases with no maternal morbidity, 6.4% of LSMM, 5.2% of PLTC, 14.5% of MNM, and 13.1% of MD also had such condition.

## 4. Discussion

The main results of this analysis showed that the overall MMR for the sample was high and over one-third of the total cases presented with any morbidity. Following some midiatic figures, this means that, for each woman who died in this sample, 262 others experienced any degree of morbidity and survived, although they may have had impairments and functioning disabilities lasting for different periods. While a minority had severe morbidity, almost one-fifth of them experienced less severe morbidity, which emphasizes the need for surveillance and timely and adequate diagnosis of complications. Apart from these main results, the study also showed that it is possible to explore a database of big birth registries in the search for variables or reported diagnosis with the specific purpose of identifying a gradient of morbidity. This would enable building a full profile of all pregnancies that could be a proxy for the theoretical continuum of morbidity, from normal pregnancy to maternal death. This gradient seems to work considering that the related adverse neonatal outcomes matched accordingly. The study also demonstrated that the history of previous pathological conditions played an important role in increasing the risk of severe maternal outcomes, and the same occurred for other factors already known to be associated, including extremes of maternal reproductive age, low literacy, absence of a steady partner, nulliparity, low number of prenatal visits, smoking, drug or alcohol use or violence, induction of labour, and elective caesarean section.

The present study has some clear limitations. It developed an operational definition for some degrees of severity on maternal morbidity with information on variables already available in a big database of an international birth registry, which was not built for that specific purpose. It is not a population-based study and does not allow generating estimates for maternal morbidity and mortality stratified for regions and countries. Unfortunately, there is no information available on the distribution of births in the settings providing information for the database. This is the main reason why data were not stratified by countries. Although the adoption of a rigorous process of checking and assuring the quality of data collection and management exists, there is still some degree of incompleteness of some information and this is also a limitation. However, this is a retrospective analysis of a database and therefore we could no longer ask for additional corrections or completeness. The amount of missing information is anyway provided for each variable assessed.

On the other hand, our study has some important strengths. To the best of our knowledge, this is the first study trying to capture a full profile of morbidity occurring during pregnancy using a routine birth registry. According to the new initiatives from WHO, this is a recommended procedure for building a full scenario of the burden of maternal morbidity for the women's lives. Using classifications for a gradient of maternal morbidity as the currently employed, additional secondary analysis will also be possible in databases from birth registries, focusing for instance on maternal morbidity linked with specific causes like hypertension or postpartum haemorrhage, twin pregnancy, obesity and overweight, ethnic groups, and other hot topics that could be easily assessed in the database, thus generating strong evidence supported by huge numbers.

The overall MMR found in this analysis was high, almost 150 per 100,000 live births. Despite the fact that recently an increased trend in the estimated MMR has been shown for the US in the last decade [14], these trends have been continuously decreasing following initiatives aligned with the United Nations Millennium Development Goals [15]. This figure is essentially the same as obtained by the biggest WHO international study focusing on maternal morbidity and mortality, which showed an MMR for the first seven days postpartum of 158 maternal deaths per 100,000 LB for 29 mainly low and middle-income countries for approximately the same period, although with important regional variations [7]. The Global Burden of Disease Study showed lower maternal mortality ratios for Latin American countries in 2013. The study evaluated 17 countries of Andean, Central, Southern, and Tropical Latin America, showing that all of them had unremitting decreases of MMR from 1990 to 2013 [16]. Such high figure reveals a still poor health and social condition of women in the region, although the figures cannot be considered as descriptive for the whole region. Although we are currently not stratifying results by country, the information that a reasonable proportion of the sample comes from low resourced settings from Latin America and the Caribbean region is of pivotal importance. Maternal mortality and morbidity depend not only on the degree of complication severity but also on the scope of health services and the quality of care provided to women. According to some studies, especially in low and middle-income settings from India, Latin America, and Africa, with important social inequalities, the disparity in the availability and use of maternal and child healthcare services contributes to the poor outcomes seen [17].

The analysis of previous morbid conditions (diabetes, hypertension, preeclampsia, eclampsia, cardiac disease, renal disease, and any other severe condition) shows a clear statement of the risks involving pregnancies with baseline complications. They presented a clear increase in the occurrence of any degree of morbidity. This highlights the importance of understanding and identifying this gradient or spectrum of complications as a way to provide appropriate management for any morbidity that could potentially develop furthermore severe complications. The Global Burden of Disease Study showed that around 70% of Latin American maternal deaths occurred in intrapartum and postpartum periods. The surveillance of maternal complications during hospitalization for childbirth might play a key role on prevention of maternal mortality [16]. Therefore, this should be translated into practice, with efforts for surveillance of clinical conditions in high-risk antenatal care, using the maternal near miss and maternal morbidity approaches as the way to interrupt the process [4].

The sociodemographic and pregnancy characteristics confirmed known risk factors for poor maternal outcomes, such as extreme age groups, nonwhite ethnicity, no stable partner, no prenatal care, smoking, drug and alcohol use, previous morbid conditions, elective C-section, or induction of labour. In addition, approximately the same results were found among cases with severe or less severe morbidity. Perhaps the best example is that of maternal age, whose extreme groups, adolescents and older women, are showed to be at higher risk of developing any degree of severity for maternal morbidity. Two big multicentre studies using the new WHO concepts and criteria for severe maternal outcomes showed similar results [5, 7]. The association between the lack of a partner support and teenage pregnancy might improve the likelihood of having a poor birth outcome. The support or involvement of a partner can affect maternal behaviour during either prenatal or perinatal periods [18]. The relationship between inadequate use of antenatal care and maternal death has already been established [19, 20]. The same set has been built by the exposure to smoking, drugs, alcohol, or violence [21, 22]. They are all known risk factors and, therefore, screening for substance abuse and violence during pregnancy should be universal, although difficult to implement, especially in low resourced settings. The recognition of all these conditions, ideally early in pregnancy, would allow stratifying women in zones of risk and the adoption of adequate interventions for care [23].

Over one-third of women displayed any maternal morbidity. This figure is quite higher than the mean 9.2% of adverse outcome index (AOI) described as a way of assessing the quality of obstetrical care [24]. This impressive finding leads to two different implications. First, it is possible to conclude that the implementation of maternal near miss concept is crucial for approaching the provision of maternal healthcare [1–4]. Second, although the minority of this 38% of women had severe morbidity, this emphasizes the lack for timely and adequate screening, diagnosis of complications, and its appropriate management and particularly for those less severe conditions that should also be addressed [10–12]. All these conditions highlight the difficulties to be overcome in order to achieve both the past United Nations' Millennium Development Goals and the current Sustainable Development Goals regarding improving maternal health condition [25]. Therefore, there is a need to reinforce international efforts to prevent maternal deaths and improve maternity care.

Neonatal outcomes matched maternal condition when considering its degree of severity. MD and MNM were clearly associated with worse perinatal outcomes and increased preterm birth rates (especially late preterm). They are due to either the severity of maternal condition itself that is able to impair the intrauterine fetal health condition and as a result of an early interruption of pregnancy because of the maternal condition, resulting in preterm birth and its negative consequences. The association of maternal complications with preterm delivery is an emerging topic in maternal and perinatal health. Provider-initiated preterm birth, for instance, is closely associated with maternal complications during pregnancy [26, 27]. Although it would be expected that worse maternal conditions are associated with worse perinatal outcomes, these results were already formally described for only one other large scale study focusing on maternal morbidity [7]. Finally, we hope that additional similar information could emerge from the analysis of other big birth registries around the world to strengthen the evidence between a close association between the degree of severity in maternal morbidity and maternal and perinatal outcomes. Hopefully, the same SIP system could in some few years to come have a more detailed analysis on these topics, now using the standardized definitions and criteria for maternal morbidity as recommended by WHO.

## 5. Conclusion

Considering the high proportion of women experiencing maternal morbidity with varying degrees of severity and the need to ensure clinical responses to these differing morbidity profiles, it is important to develop and sustain active surveillance systems that would theoretically follow women in real time while they are experiencing the process of pregnancy and childbirth and possibly some related complications. Following the WHO recommendation, the SIP birth registry recently adopted the same concepts and criteria for maternal near miss [1, 4]. Then we hope that in a near future we can be able not only to show updated data prospectively collected on severity of maternal morbidity but also to report the experience of an international birth registry working as an online surveillance system for maternal mortality and morbidity in Latin American and Caribbean settings, the CLAP-Network [28].

## Figures and Tables

**Box 1:**
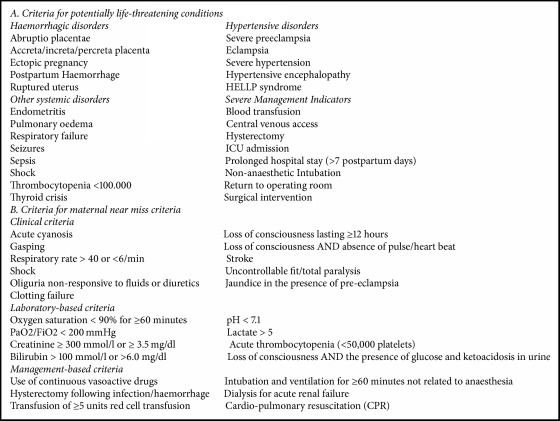
The WHO criteria for potentially life-threatening conditions and maternal near miss (modified from [1]).

**Table 1:** Prevalence of adverse maternal outcomes and correspondent health indicators. CLAP 2009–2012.

Maternal outcomes	*N*	%	Health indicators
MD	1,028	0.14	MMR = 147.3/100,000 LB
MNM	21,985	3.1	MNMR = 31.5/1000 LB
*SMO*	*23,013*	*3.2*	*SMOR = 33.0/1000 LB*
PLTC	110,038	15.5	PLTCR = 157.7/1000 LB
LSMM	137,589	19.3	LSMMR = 197.2/1000 LB
AMM	270,640	38.0	AMMR = 387.8/1000 LB
No morbidity	441,441	62.0	

Total	712,081	100.0	Total LB: 697,820

AMM: any maternal morbidity; AMMR: any maternal morbidity ratio; LB: live births; LSMM: less severe maternal morbidity; LSMMR: less severe maternal morbidity ratio; MD: maternal death; MMR: maternal mortality ratio; MNM: maternal near miss; MNMR: maternal near miss ratio; PLTC: potentially life-threatening condition; PLTCR: potentially life-threatening condition ratio; SMO: severe maternal outcome; SMOR: severe maternal outcome ratio.

**Table 2:** Prevalence of some previous pathological maternal conditions according to the type of adverse maternal outcomes. CLAP 2009–2012.

Previous conditions	MD	MNM	PLTC	LSMM	Any (AMM)	No morbidity	*p* value^*∗*^
Diabetes^a^	8 (0.8)	230 (1.1)	861 (0.8)	1681 (1.3)	2780 (1.1)	1043 (0.2)	<.001
Hypertension^b^	32 (3.2)	952 (4.5)	1971 (1.8)	2310 (1.7)	5265 (2.0)	4704 (1.1)	<.001
Preeclampsia^c^	27 (2.7)	730 (3.4)	1403 (1.3)	2136 (1.6)	4296 (1.6)	3750 (0.9)	<.001
Eclampsia^d^	12 (1.2)	125 (0.6)	176 (0.2)	213 (0.3)	526 (0.2)	594 (0.1)	<.001
Other severe conditions^e^	26 (2.6)	873 (4.1)	5014 (4.7)	3603 (2.7)	9516 (3.6)	5730 (1.3)	<.001
Cardiac disease^f^	7 (0.7)	227 (1.1)	194 (0.2)	180 (0.1)	608 (0.2)	374 (0.1)	<.001
Renal disease^g^	1 (0.1)	105 (0.5)	173 (0.2)	141 (0.1)	420 (0.2)	274 (0.1)	<.001
Any previous condition^h^	90 (9.1)	2542 (13.0)	8723 (8.3)	8834 (10.4)	20189 (9.6)	14493 (3.5)	<.001

^*∗*^Pearson Chi-square test. Missing information for a: 2.8%; b: 2.7%; c: 2.8%; d: 11.3%; e: 3.6%; f: 3.4%; g: 3.5%; h: 12.4% of cases; AMM: any maternal morbidity; LSMM: less severe maternal morbidity; MD: maternal death; MNM: maternal near miss; PLTC: potentially life-threatening condition.

**Table 3:** Crude (PR) and adjusted (APR) estimated risks of maternal death (MD) and of maternal near miss (MNM) according to some maternal and obstetric characteristics. CLAP 2009–2012.

Characteristics	No morbidity	MD	PR (95% CI)	APR (95% CI)	MNM	PR (95% CI)	APR (95% CI)
Maternal age (years)^a^							
10–19	108,907	276	1.15 [0.89–1.48]	1.43 [0.87–2.35]	5,689	**1.21 [1.14–1.27]**	**1.13 [1.05–1.21]**
20–24	128,968	285	Ref.	Ref.	5,530	Ref.	Ref.
25–29	96,493	187	0.88 [0.74–1.04]	**0.79 [0.63–0.98]**	4,433	1.07 [0.98–1.16]	1.06 [0.97–1.17]
30–34	64,458	169	1.19 [0.89–1.58]	0.79 [0.61–1.02]	3,432	**1.23 [1.12–1.35]**	1.12 [0.95–1.32]
35–55	40,585	109	1.21 [0.67–2.19]	0.72 [0.37–1.40]	2,811	**1.58 [1.40–1.78]**	**1.39 [1.04–1.87]**

Ethnicity/skin colour^b^							
White	91,116	175	Ref.	Ref.	6,556	Ref.	Ref.
Mixed	298,612	231	0.40 [0.11–1.42]	**0.35 [0.13–0.98]**	12,082	0.58 [0.29–1.14]	0.70 [0.47–1.04]
Others	19,328	594	**15.55 [4.66–51.96]**	**18.95 [8.01–44.86]**	1,749	1.24 [0.76–2.00]	1.09 [0.84–1.42]

Literacy^c^							
No or primary	203,185	379	0.68 [0.41–1.12]	0.90 [0.58–1.37]	8,418	**0.73 [0.62–0.85]**	0.96 [0.79–1.16]
Secondary or university	217,307	600	Ref.	Ref.	12,616	Ref.	Ref.

Marital status^d^							
Married + stable part	364,380	825	Ref.	Ref.	17,369	Ref.	Ref.
Single + other	56,037	142	1.12 [0.66–1.89]	0.87 [0.64–1.18]	3,695	**1.36 [1.10–1.68]**	**1.23 [1.09–1.38]**

Parity^e^							
Nullipara	153,659	350	0.93 [0.71–1.23]	0.64 [0.36–1.12]	9,963	**1.50 [1.37–1.65]**	**1.32 [1.16–1.50]**
Multipara	250,299	612	Ref.	Ref.	10,577	Ref.	Ref.

Any previous condition^f^							
Yes	14,493	90	**2.75 [1.20–6.29]**	**2.29 [1.53–3.41]**	2,542	**3.63 [2.39–5.52]**	**2.49 [1.88–3.30]**
No	398,396	897	Ref.	Ref.	17,059	Ref.	Ref.

Number of prenatal care visits^g^							
0	30,445	60	0.84 [0.47–1.51]	1.05 [0.52–2.11]	1,910	1.22 [0.85–1.74]	**1.52 [1.23–1.87]**
1–4	115,059	328	1.21 [0.91–1.62]	1.00 [0.81–1.22]	5,623	0.96 [0.79–1.18]	**1.21 [1.02–1.45]**
>4	252,273	592	Ref.	Ref.	12,822	Ref.	Ref.

Smoking, drugs, alcohol, or violence^h^							
Yes	30,559	76	0.95 [0.16–5.46]	0.87 [0.48–1.58]	2,255	**1.70 [1.07–2.70]**	**1.18 [1.11–1.26]**
No	288,779	760	Ref.	Ref.	12,176	Ref.	Ref.

Previous C-section^i^							
Yes	50,662	149	1.27 [0.98–1.63]	0.73 [0.46–1.18]	2,996	1.12 [0.94–1.34]	**0.58 [0.51–0.65]**
No	303,471	705	Ref.	Ref.	15,929	Ref.	Ref.

Onset of labour^j^							
Spontaneous	340,966	627	Ref.	Ref.	12,910	Ref.	Ref.
Induced	20,578	66	1.74 [0.70–4.34]	**1.66 [1.22–2.26]**	1,436	**1.79 [1.52–2.11]**	1.20 [0.89–1.62]
Elective C-section	50,673	289	**3.09 [1.63–5.85]**	**1.94 [1.42–2.66]**	6,408	**3.08 [2.28–4.14]**	**1.63 [1.17–2.27]**

Mode of delivery^k^							
C-section	123,423	447	**1.99 [1.34–2.95]**	1.19 [0.94–1.50]	12,877	**3.40 [2.80–4.14]**	**2.37 [1.86–3.01]**
Vaginal (any)	310,183	563	Ref.	Ref.	8,864	Ref.	Ref.

Total	441,441	1028			21,985		

Missing information for a: 0.4%; b: 6.9%; c: 4.1%; d: 4.2%; e: 7.6%; f: 12.4%; g: 7.6%; h: 31.1%; i: 16.8%; j: 5.4%; k: 1.2% of cases; APR: adjusted prevalence ratio (adjusted for cluster effect and all other predictors); MD: maternal death; MNM: maternal near miss.

**Table 4:** Crude (PR) and adjusted (APR) estimated risks of potentially life-threatening condition (PLTC) and of less severe maternal morbidity (LSMM) according to some maternal and obstetric characteristics. CLAP 2009–2012.

Characteristics	No morbidity	PLTC	PR (95% CI)	APR (95% CI)	LSMM	PR (95% CI)	APR (95% CI)
Maternal age (years)^a^							
10–19	108,907	25468	1.03 [0.92–1.15]	1.05 [0.93–1.19]	35482	1.04 [0.97–1.11]	**1.10 [1.02–1.18]**
20–24	128,968	29098	Ref.	Ref.	40029	Ref.	Ref.
25–29	96,493	24018	1.08 [1.00–1.18]	**1.05 [1.02–1.08]**	28105	0.95 [0.85–1.07]	**0.93 [0.89–0.97]**
30–34	64,458	18924	**1.23 [1.06–1.43]**	**1.12 [1.05–1.18]**	19737	0.99 [0.89–1.10]	**0.92 [0.85–0.99]**
35–55	40,585	12150	**1.25 [1.11–1.42]**	**1.18 [1.10–1.26]**	13714	1.07 [0.94–1.21]	1.00 [0.95–1.06]

Ethnicity/skin colour^b^							
White	91,116	48,646	Ref.	Ref.	38,882	Ref.	Ref.
Mixed	298,612	48,499	**0.40 [0.17–0.94]**	0.44 [0.19–1.01]	75,497	0.67 [0.40–1.15]	**0.57 [0.41–0.78]**
Others	19,328	5,790	0.66 [0.39–1.12]	0.83 [0.54–1.27]	15,303	1.48 [0.97–2.25]	0.99 [0.67–1.47]

Literacy^c^							
No or primary	203,185	40,699	0.71 [0.50–1.02]	**0.83 [0.74–0.94]**	48,548	0.69 [0.45–1.05]	0.90 [0.74–1.09]
Secondary or university	217,307	66,638	Ref.	Ref.	84,547	Ref.	Ref.

Marital status^d^							
Married + stable part	364,380	89,465	Ref.	Ref.	110,699	Ref.	Ref.
Single + other	56,037	17,616	1.21 [0.99–1.48]	1.04 [0.93–1.15]	22,171	1.22 [0.97–1.53]	1.10 [1.00–1.22]

Parity^e^							
Nullipara	153,659	43,923	**1.20 [1.09–1.33]**	**1.14 [1.06–1.23]**	56,559	**1.16 [1.04–1.30]**	0.95 [0.80–1.13]
Multipara	250,299	56,737	Ref.	Ref.	75,554	Ref.	Ref.

Any previous condition^f^							
Yes	14,493	8,723	**1.93 [1.29–2.90]**	**1.43 [1.09–1.87]**	8,834	**2.36 [1.55–3.58]**	**1.52 [1.35–1.72]**
No	398,396	96,144	Ref.	Ref.	76,313	Ref.	Ref.

Number of prenatal care visits^g^							
0	30,445	6,820	0.78 [0.31–1.99]	0.99 [0.66–1.50]	9,831	0.95 [0.56–1.60]	**0.76 [0.61–0.95]**
1–4	115,059	22,285	0.69 [0.39–1.22]	0.87 [0.73–1.02]	35,221	0.91 [0.71–1.17]	0.92 [0.79–1.06]
>4	252,273	77,050	Ref.	Ref.	87,566	Ref.	Ref.

Smoking, drugs, alcohol, or violence^h^							
Yes	30,559	17,576	1.76 [0.84–3.72]	1.01 [0.89–1.14]	14,097	**2.19 [1.37–3.50]**	**1.28 [1.15–1.42]**
No	288,779	75,450	Ref.	Ref.	48,689	Ref.	Ref.

Previous C-section^i^							
Yes	50,662	8,748	**0.68 [0.57–0.82]**	**0.76 [0.68–0.85]**	19,847	1.09 [0.97–1.21]	0.89 [0.80–1.00]
No	303,471	83,961	Ref.	Ref.	106,305	Ref.	Ref.

Onset of labour^j^							
Spontaneous	340,966	87,326	Ref.	Ref.	97,812	Ref.	Ref.
Induced	20,578	10,826	**1.69 [1.34–2.13]**	1.13 [0.87–1.47]	9,894	**1.46 [1.25–1.69]**	**1.39 [1.12–1.73]**
Elective C-section	50,673	9,082	0.75 [0.47–1.18]	**0.66 [0.46–0.94]**	24,656	**1.47 [1.16–1.86]**	**1.33 [1.12–1.58]**

Mode of delivery^k^							
C-section	123,423	27,657	0.88 [0.66–1.18]	0.83 [0.68–1.02]	50,768	**1.35 [1.12–1.62]**	**1.16 [1.05–1.28]**
Vaginal (any)	310,183	81,745	Ref.	Ref.	85,770	Ref.	Ref.

Total	441,441	110,038			137,589		

Missing information for a: 0.4%; b: 6.9%; c: 4.1%; d: 4.2%; e: 7.6%; f: 12.4%; g: 7.6%; h: 31.1%; i: 16.8%; j: 5.4%; k: 1.4% of cases; APR: adjusted prevalence ratio (adjusted for cluster effect and all other predictors); LSMM: less severe maternal morbidity; PLTC: potentially life-threatening condition.

**Table 5:** Neonatal outcomes according to the adverse maternal outcomes. CLAP 2009–2012.

Neonatal outcome	MD	MNM	PLTC	LSMM	Any (AMM)	No morbidity	*p* value^*∗*^
Gestational age at birth^a^							**0.007**
<32 weeks	68 (6.9)	1438 (7.0)	3,125 (3.0)	5,116 (3.9)	9,747 (3.8)	10,620 (2.6)	
32–36	141 (14.2)	4,114 (20.0)	8,978 (8.5)	13,049 (9.9)	26,282 (10.1)	28,853 (7.0)	
≥37 weeks	783 (78.9)	14,985 (73.0)	93,534 (88.5)	113,707 (86.2)	223,009 (86.1)	369,957 (90.4)	

Birth weight^b^							**<0.001**
<2500 g	181 (18.0)	5,364 (24.8)	9,135 (8.4)	14,786 (10.9)	29,466 (11.1)	35,103 (8.1)	
2500–3999 g	755 (75.0)	15,451 (71.4)	93,650 (86.4)	113,928 (84.3)	223,784 (84.1)	378,050 (87.6)	
≥4000 g	71 (7.1)	822 (3.8)	5,571 (5.1)	6,495 (4.8)	12,959 (4.8)	18,218 (4.2)	

Apgar 5th min <7^c^	49 (5.0)	789 (3.7)	2,105 (2.0)	2,319 (1.7)	5,262 (2.0)	7,363 (1.7)	0.088
Vital status at birth							0.726
Alive	986 (95.1)	21,431 (97.5)	107,788 (98.0)	134,556 (97.8)	264,761 (97.8)	433,059 (98.1)	
Fetal death	42 (4.9)	554 (2.5)	2,250 (2.0)	3,033 (2.2)	5,879 (2.2)	8,382 (1.9)	

Child condition at maternal discharge^d^							
Alive	736 (95.9)	16,906 (96.7)	97,206 (98.4)	112,886 (98.2)	227,734 (98.1)	365,715 (99.0)	**0.027**
Neonatal death	28 (3.6)	358 (2.0)	804 (0.8)	915 (0.8)	2,105 (0.9)	2,066 (0.6)	
Referred	4 (0.5)	227 (1.3)	824 (0.8)	1,144 (1.0)	2,199 (1.0)	1,792 (0.5)	

Neonatal near miss^**∗∗**e^	128 (13.1)	2,964 (14.5)	5,516 (5.2)	8,440 (6.4)	17,048 (6.6)	19,757 (4.9)	**0.008**

^**∗**^Pearson Chi-square test corrected by the cluster design effect. ^**∗∗**^Neonatal near miss: birthweight < 1750 g or Apgar 5th min <7 or GA <33 weeks; missing information for a: 6.1%; b: 2.0%; c: 3.0%; d: 15.5%; e: 6.5% of cases; AMM: any maternal morbidity; LSMM: less severe maternal morbidity; MD: maternal death; MNM: maternal near miss; PLTC: potentially life-threatening condition.

## Abbreviations

AMM: Any maternal morbidity
AMMR: Any maternal morbidity ratio
AOI: Adverse outcome index
CLAP-WR: Latin American Center for Perinatology, Women and Reproductive Health
ICU: Intensive care unit
LB: Live births
LSMM: Less severe maternal morbidity
LSMMR: Less severe maternal morbidity ratio
MD: Maternal death
MMR: Maternal mortality ratio
MNM: Maternal near miss
MNMR: Maternal near miss ratio
PAHO: Pan American Health Organization
PLTC: Potentially life-threatening condition
PLTCR: Potentially life-threatening condition ratio
SIP: Perinatal Information System
SMO: Severe maternal outcome
SMOR: Severe maternal outcome ratio
WHO: World Health Organization.
